# Why Social Pain Can Live on: Different Neural Mechanisms Are Associated with Reliving Social and Physical Pain

**DOI:** 10.1371/journal.pone.0128294

**Published:** 2015-06-10

**Authors:** Meghan L. Meyer, Kipling D. Williams, Naomi I. Eisenberger

**Affiliations:** 1 Psychology Department, University of California Los Angeles, Los Angeles, California, United States of America; 2 Department of Psychology, Purdue University, West Lafayette, Indiana, United States of America; University of Udine, ITALY

## Abstract

Although social and physical pain recruit overlapping neural activity in regions associated with the affective component of pain, the two pains can diverge in their phenomenology. Most notably, feelings of social pain can be re-experienced or “relived,” even when the painful episode has long passed, whereas feelings of physical pain cannot be easily relived once the painful episode subsides. Here, we observed that reliving social (vs. physical) pain led to greater self-reported re-experienced pain and greater activity in affective pain regions (dorsal anterior cingulate cortex and anterior insula). Moreover, the degree of relived pain correlated positively with affective pain system activity. In contrast, reliving physical (vs. social) pain led to greater activity in the sensory-discriminative pain system (primary and secondary somatosensory cortex and posterior insula), which did not correlate with relived pain. Preferential engagement of these different pain mechanisms may reflect the use of different top-down neurocognitive pathways to elicit the pain. Social pain reliving recruited dorsomedial prefrontal cortex, often associated with mental state processing, which functionally correlated with affective pain system responses. In contrast, physical pain reliving recruited inferior frontal gyrus, known to be involved in body state processing, which functionally correlated with activation in the sensory pain system. These results update the physical-social pain overlap hypothesis: while overlapping mechanisms support live social and physical pain, distinct mechanisms guide internally-generated pain.

## Introduction

“Moral wounds have this peculiarity—they may be hidden, but they never close; always painful, always ready to bleed when touched, they remain fresh and open in the heart.”

-Alexandre Dumas, “The Count of Monte Cristo”

Alexandre Dumas highlights a curious part of life: moral wounds, whether a result of being wronged, betrayed, or excluded, do not easily heal. Instead, they are readily re-experienced, often with very little effort. The tendency to re-experience pain long after a negative social event occurred has been documented empirically as well. A close other’s betrayal occurring years ago continues to plague older adults [1], the distress of childhood bullying persists into young adulthood [2], and even briefly writing about a former negative social experience leads to an intense reliving of the pain that occurred up to five years prior [3]. In contrast, former physical pain is difficult to relive. Although people are able to retrieve physical pain memories (remembering the qualities of the pain; [4, 5]), they are less able to re-experience that pain once the painful episode subsides [3, 5].

The dichotomy in the capacity to relive social and physical pain is interesting given what is known about how the brain processes these two forms of suffering. Negative social experiences, such as exclusion [6], romantic rejection [7], and negative social feedback [8] rely on the same neural system supporting the affective or ‘unpleasant’ component of physical pain (dorsal anterior cingulate cortex (dACC) and anterior insula (AI)), hence the coining of the term, ‘social pain’ [6] (it has also been suggested that these regions play a more general role in processing salience; [9]). If the same affective pain system is recruited during live social and physical pain, then why would social pain be more easily relived than physical pain?

One possibility is that, while social and physical pains both activate the affective pain system during live experiences of pain, social pain may preferentially activate the affective pain system during reliving. Consistent with this suggestion, thinking about a former experience of social pain (e.g., romantic rejection) activates the affective pain system [7], whereas keeping in mind a representation of former physical pain activates the sensory-discriminative pain system, but not the dACC, often associated with the affective component of pain [10, 11]. Thus, preferentially activating affective pain regions, particularly dACC, during relived social versus physical pain may contribute to the phenomenology of greater relived social versus physical pain.

If reliving social pain preferentially recruits the affective pain system, a corollary question is why does this difference occur during reliving? An answer to this question may stem from the fact that reliving past pain requires inducing pain without the presence of direct noxious input. Thus, individuals may recruit different top-down neurocognitive mechanisms to induce past social and physical pain, and these mechanisms may differentially relate to affective pain and sensory pain system responding. Indeed, portions of the pain system, particularly dACC, have been shown to differentially communicate with other brain structures depending on the type of pain experienced [12]. Reliving social pain may engage social cognitive processing (e.g., why an ex-partner wronged you) which recruits a medial frontoparietal network, particularly dorsal medial prefrontal cortex (DMPFC/Brodmann area 8/9; [13–15]). Interestingly, although not typically studied in the context of affective processes, DMPFC has been shown to be involved in increasing negative affective responses [16, 17] and has a strong functional relationship with the affective pain system when participants must consider others’ mental states to induce negative emotions. For example, DMPFC and affective pain regions (dACC, AI) parametrically increase as participants feel worse in response to thinking about how other people perceive them [8] and functionally correlate when considering another person’s state of mind to vicariously feel their suffering during empathy [18]. Moreover, in animals, stimulation of a region analogous to the human DMPFC enhances the expression of negative affective responses during fear conditioning [19], suggesting a causal role for DMPFC in increasing negative affective responses. Thus, DMPFC, which may be recruited during the mental state processing associated with social pain reliving, may communicate with the affective pain system to facilitate reliving the affective component of social pain.

In contrast, when reliving physical pain, individuals may focus on the bodily states related to the pain (e.g., the location and sensation of soreness associated with a broken limb) and this form of body state processing recruits a lateral frontoparietal system [20], particularly inferior frontal gyrus (IFG; [21–23]). IFG activates in response to feeling and perceiving physical attributes, from limb sensation [22] to voice and body recognition [20, 24] and is involved in retrieving information about bodily states [25, 26], including physical pain memory retrieval [27]. Important to the phenomenon of reduced self-reported pain during physical pain reliving, IFG does not seem to enhance affective pain system activity during pain experience. In fact, several studies have shown the opposite, namely that activation in this region reduces self-reported physical pain [28] and social pain [6], and is associated with decreased affective pain system activation during these processes [6, 28].

Taken together, it is possible that social pains are more easily relived because they preferentially engage the affective pain system during reliving. This preferential affective pain system responding may be due, in part, to the recruitment of different prefrontal pathways to relive former social and physical pain. Reliving past social pain may involve more cognition dedicated to the mental states of others and this may activate DMPFC, which may functionally communicate with the affective pain system during social pain reliving. In contrast, reliving past physical pain may involve more cognition about the physical states of the body and recruit IFG, which may communicate with the sensory pain system in attempt to bring sensory qualities of a former pain to mind. To test these predictions, participants underwent functional magnetic resonance imaging (fMRI) while they relived memories of social and physical pain. Prior to their scan, participants completed journal entries detailing their memories and rated how much pain they experienced at the time of the event (initial pain). During their fMRI scanner session, participants rated how much pain they experienced upon reliving the pain in the scanner (relived pain).

## Methods

### Participants

Eighteen right-handed individuals (8 male, 10 female; mean age = 22.8 years, SD = 2.9 years) participated in the study. Ethnic identification of the participants were as follows: 61% Caucasian, 11% Asian American, 17% Latino/a, 11% other. Participants were recruited if they met the qualification of having experienced both a very bad socially painful event (e.g., break-up from a romantic relationship, exclusion from a friend or family member, or some kind of betrayal, etc.) and a very bad physically painful event (e.g., broken bone, hospitalization, physical accident, etc.) in the past five years. To ensure MRI compatibility and facilitate interpretable neural results, participants were right-handed, without metal in their body, not taking psychiatric medication, spoke English as their native language, not claustrophobic, and not pregnant. The UCLA Institutional Review Board (UCLA IRB) approved this study (approval number: IRB#11–003017). All participants provided written consent in accordance with the UCLA Institutional Review Board.

### Procedure

Prior to the fMRI scan, participants completed an online questionnaire in which they wrote journal entries describing their social pain memory and physical pain memory, as well as a neutral, non-painful social memory (e.g., watching a movie with a friend) and a neutral, non-painful physical memory (e.g., a walk they took to get to campus). Prior to writing each entry, participants rated on a 0 to 10 scale how much pain they felt at the time of the event. Following past research on reliving social pain [3], to eliminate carry-over effects in pain ratings and writing experiences between the physical and social pain questions, in between these sections of the online questionnaire participants completed a visuospatial task, in which they indicated which of two shapes matched a target shape.

**Fig 1:**
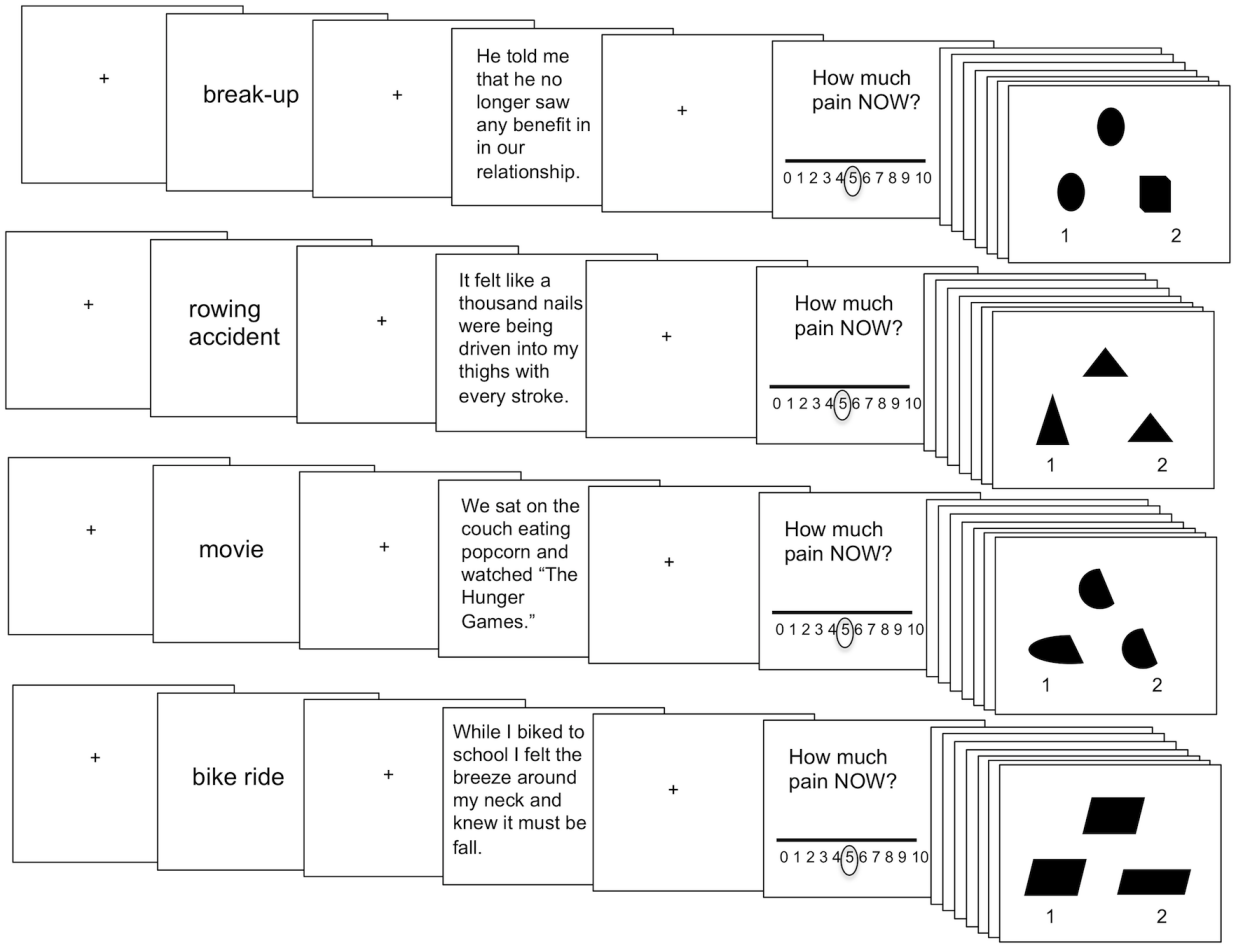
Reliving task schematic. Each reliving block began with 7 seconds of fixation. Next, participants observed a cue for 2 seconds indicating which memory they were about to relive. Then, after a 1 second fixation screen participants had 5 seconds to read an excerpt taken from their memory journal to facilitate their reliving. Next, participants had 15 seconds of fixation during which time they were instructed to relive their memory. Participants then used a 0–10 sliding scale (which originally appeared at rating 5) to rate how much pain they felt in the scanner. Reliving was followed by 18 seconds of completing visuospatial match to sample trials (9 match to sample trials, each shown for 2 seconds) to avoid carry over effects between memories.

During the fMRI scanning session, participants performed a computerized task in which they relived the memories they described in their online questionnaire (Fig 1). Prior to each reliving block, participants observed a fixation crosshair for seven seconds. After fixation, participants were shown a phrase indicating which memory they were about to relive (‘memory cue’; 2 seconds). Memory cues were brief indications of the subject’s memory, such as: ‘break-up’; ‘rowing accident’; ‘movie’; ‘bike ride.’ Next, after 1 second of fixation, a short statement describing the memory (taken directly from their journal entry) appeared on the screen prior to reliving (5 seconds). Participants then had 15 seconds to relive the memory, during which time there was a fixation crosshair on the computer screen. Participants were told that during reliving they should try to re-experience the event as though it were happening in the present moment and to re-experience their feelings, sensations, thoughts, and images. After reliving the memory, participants rated from 0–10 how much pain they felt during the reliving. Because some participants closed their eyes during reliving, a beep sounded after the 15-second reliving block to notify participants to open their eyes so that they could make their pain rating on the following screen.

The reliving task comprised two runs and each block (social pain; physical pain; social neutral; physical neutral) appeared twice per run. Block order was counter-balanced across participants. Again, consistent with past research on reliving social pain [3], to eliminate carry-over effects in pain reliving, after each reliving block participants completed nine visuospatial task trials (2 seconds/trial; 18 seconds total) in which they indicated which of two shapes matched a target shape. Stimuli were presented using the MATLAB (The MathWorks, Inc., Natick, MA, USA) Psychophysics Toolbox (version 3.0.9; [29]). Participants viewed stimuli through LCD goggles (800 x 600 pixels) and made their sliding scale pain ratings with a button-box.

To examine the extent to which participants’ social and physical pain memories engaged mental state and bodily state processing, after the scan session, eight raters (who did not interact with the study participants) judged participants’ social and physical pain memory journal entries along these two dimensions. To assess mental state processing within the journal entries, raters answered the question: “To what extent was this person thinking about other peoples’ thoughts, feelings, or intentions?” and “To what extent was this person thinking about his or her own thoughts, feelings or intentions?” These two items were highly correlated in both the social pain and physical pain conditions (r social = .71, *p* = .001; r physical = .47, *p*<.05), and so the average of these two ratings was computed to make a composite mental state processing score. To assess body state processing within the journal entries, raters answered the question, “To what extent was this person thinking about the physical sensations (e.g., nausea, tingling, soreness) and/or physical states of their body (e.g., blood, bruising)?” Raters made their ratings using a 7-point scale (1 = not at all to 7 = very much). For each journal entry, the eight raters’ ratings were averaged as a composite score.

### fMRI Data Acquisition

FMRI images were collected on a Siemens Trio 3-Tesla MRI scanner. Functional T2*-weighted echoplanar image volumes (EPIs; slice thickness = 3 mm, gap = 1 mm, 36 slices, TR = 2000 ms, TE = 25ms, flip angle = 90°, matrix = 64x64, FOV = 200mm) were acquired during each reliving scan. Two structural scans were acquired for data preprocessing: a T2-weighted matched-bandwidth anatomical scan (same parameters as EPIs, except: TR = 5000 ms, TE = 34 ms, flip angle = 90°, matrix = 128 x 128) and a T1-weighted magnetization-prepared rapid-acquisition gradient echo anatomical scan (slice thickness = 1 mm, 176 slices, TR = 2530 ms, TE = 3.31 ms, flip angle = 7°, matrix = 256 x 256, FOV = 256 mm).

### fMRI Data Analysis

Statistical Parametric Mapping (SPM8, Wellcome Department of Cognitive Neurology, London, UK) was used to analyze functional images. The following preprocessing steps were performed to prepare the fMRI data for statistical analysis. First, each EPI volume was realigned to the first EPI volume of each run. Second, the T1 structural volume was co-registered to the mean EPI. Third, to normalize the T1 structural volume to a common group-specific space (with subsequent affine registration to MNI space), we used the group-wise DARTEL registration method included in SPM8 [30]. Fourth, we normalized the EPI volumes to MNI space using the deformation flow fields generated in the previous step, which simultaneously re-sampled volumes (3mm isotropic) and applied spatial smoothing (Gaussian kernel of 8mm, full width at half maximum).

At the first level of analysis, each subject’s preprocessed data was submitted to a general linear model in which we modeled regressors for each condition of interest (reliving social pain; reliving physical pain; reliving social neutral; reliving physical neutral) and regressors of no interest capturing the portions of the task not related to reliving as well as 6 motion regressors for each of the motion parameters from image realignment. At this first level of analysis, our contrasts modeled the main effects of each reliving condition as 20-second blocks (using a canonical (double-gamma) hemodynamic response function for convolution) beginning at the onset of the reliving cue and ending at the offset of the 15-second fixation period.

Next, subjects’ first level contrasts were brought to a second level full factorial design to test our hypotheses regarding reliving social pain (vs. social neutral), reliving physical pain (vs. physical neutral), and the difference between these two forms of reliving. Several studies have isolated the neural regions associated with social and physical pain and we had specific hypotheses that the affective pain system and DMPFC would be associated with social pain reliving, while the sensory pain system and IFG would be associated with physical pain reliving. We therefore interrogated our second level analyses within two anatomical masks based on past work and our hypotheses: 1) a mask that included the DMPFC, dACC, and AI and 2) a mask that included the IFG, somatosensory cortices (S1 and S2) and posterior insula (PI).

For regions in the affective pain system (dACC and AI) and sensory pain system (S1, S2, and posterior insula (PI)), we constructed regions of interest (ROIs) in PickAtlas [31] using templates from the atlas of Tzourio-Mazoyer et al. [32]. Although the regions involved in the affective vs. sensory components of pain can not be completely dissociated, we use this general categorization based on prior lesion studies highlighting a more dominant role of the dACC and AI in the affective component of pain [52–54] and S1, S2, and PI in the sensory component of pain [55, 56] (as well as prior reviews of the neural correlates of pain processing (e.g., [57]). The dACC ROI combined Brodmann Areas 24 and 32 and used a rostral boundary of y = +36 on the basis of criteria established by Vogt et al. [33] and a caudal boundary of y = 16 on the basis of summary data indicating that the majority of physical pain study activations occur anterior to that coordinate [34]. To create ROIs for the anterior and posterior insula, the insula was divided into thirds (to account for anterior insula, middle insula, and posterior insula (y = -32 to 11 for posterior insula; y = 10 to 32 for anterior insula)), which correspond with functional and anatomical boundaries observed in primates, including humans [35]. The S1 ROI comprised the summation of Brodmann Areas 1, 2, and 3. The S2 ROI comprised Brodman Area 40 and the rolandic operculum bounded by coordinates drawn from anatomical boundaries defined by Caspers et al. [36] and Eickhoff et al. [37] (y = -16 to -36 and z = 16 to 36).

Because the DMPFC and IFG are very large structures and less well anatomically defined than regions comprising the pain systems, we created spheres (each with an 8 mm radius) around previously reported coordinates that observed these regions in relevant studies. For the DMPFC ROI, the coordinate was taken from Spunt et al., (2012), which identified a cluster with the peak (-6 59 22) in a conjunction analysis of both visual and verbal stimuli that require subjects to determine people’s intentions. For the IFG ROI, the coordinate was taken from Fairhurst et al. [11] which identified a cluster with the peak (-42 40 6) in a conjunction analysis of both live physical pain and memory for physical pain.

Individual ROIs were then merged to create: 1) an ‘affective pain mask’ which consisted of the dACC, AI, and DMPFC ROIs and 2) a ‘sensory pain mask’ which consisted of the S1, S2, PI, and IFG ROIs (See S1 Fig). We then used AlphaSim in AFNI [38] to determine a joint voxelwise and cluster-size threshold that corresponded to a false-positive discovery rate of 5% across each mask as estimated by Monte Carlo simulation (10,000 iterations). Based on these estimations, analyses interrogated in the affective pain mask used a threshold of p<.005, 10 voxels and analyses interrogated in the sensory pain mask used a threshold of p<.005, 16 voxels.

To examine which conditions explained observed interaction effects, we extracted cluster parameter estimates from contrasts that separately modeled each condition versus implicit baseline. Post-hoc statistical tests of simple effects were then tested in SPSS software. The purpose of the post-hoc analyses was to reveal which differences between conditions drive the observed interaction effects, but the significance values should be interpreted with caution [39]. In addition, a regression analysis of self-reported pain while reliving social vs. physical pain was conducted on a second-level t-contrast comparing social pain reliving to physical pain reliving. Finally, masked results were followed up with whole-brain analyses using a threshold of p<.005, 10 voxels. This more liberal whole-brain threshold was used to explore the possibility that other regions besides those hypothesized were associated with reliving social and physical pain.

### Psychophysiological Interaction Analyses (PPI)

To test how DMPFC and IFG activity communicate with other neural regions during reliving, we performed psychophysiological interaction analyses (PPI; [40]). PPI analysis identifies brain regions in which neural activity correlates more strongly with a predefined ‘seed’ region (here, DMPFC and IFG) during one condition compared to another (here, social pain reliving relative to social neutral reliving and physical pain reliving relative to physical neutral reliving). PPI analysis was performed using the SPM generalized PPI toolbox (http://www.martinos.org/&mclaren/ftp/Utilities_DGM). We used the cluster of DMPFC activation observed in the social pain reliving vs. physical pain reliving contrast as our seed region with the rationale that this DMPFC activation distinguishes social pain reliving from physical pain reliving while still being independent of the direct contrast of social pain reliving vs. social neutral reliving. At the individual subject level, we extracted a deconvolved time course averaged across the voxels in our DMPFC seed. This time course was then included in a generalized PPI model, together with a psychological regressor and a PPI regressor for each of the conditions of interest (reliving social pain, reliving social neutral). Resulting PPI connectivity estimates were taken to the group level, where we examined neural regions within our two anatomical masks that were correlated with the timecourse of activity in the DMPFC seed during reliving social pain versus reliving social neutral. To examine which neural regions’ activation is coordinated with IFG during physical pain reliving, we also performed PPI analysis on the physical pain vs. physical neutral reliving with an IFG cluster observed during physical pain reliving vs. social pain reliving as a seed.

## Results

### Reliving socially painful memories leads to more re-experienced pain

**Fig 2:**
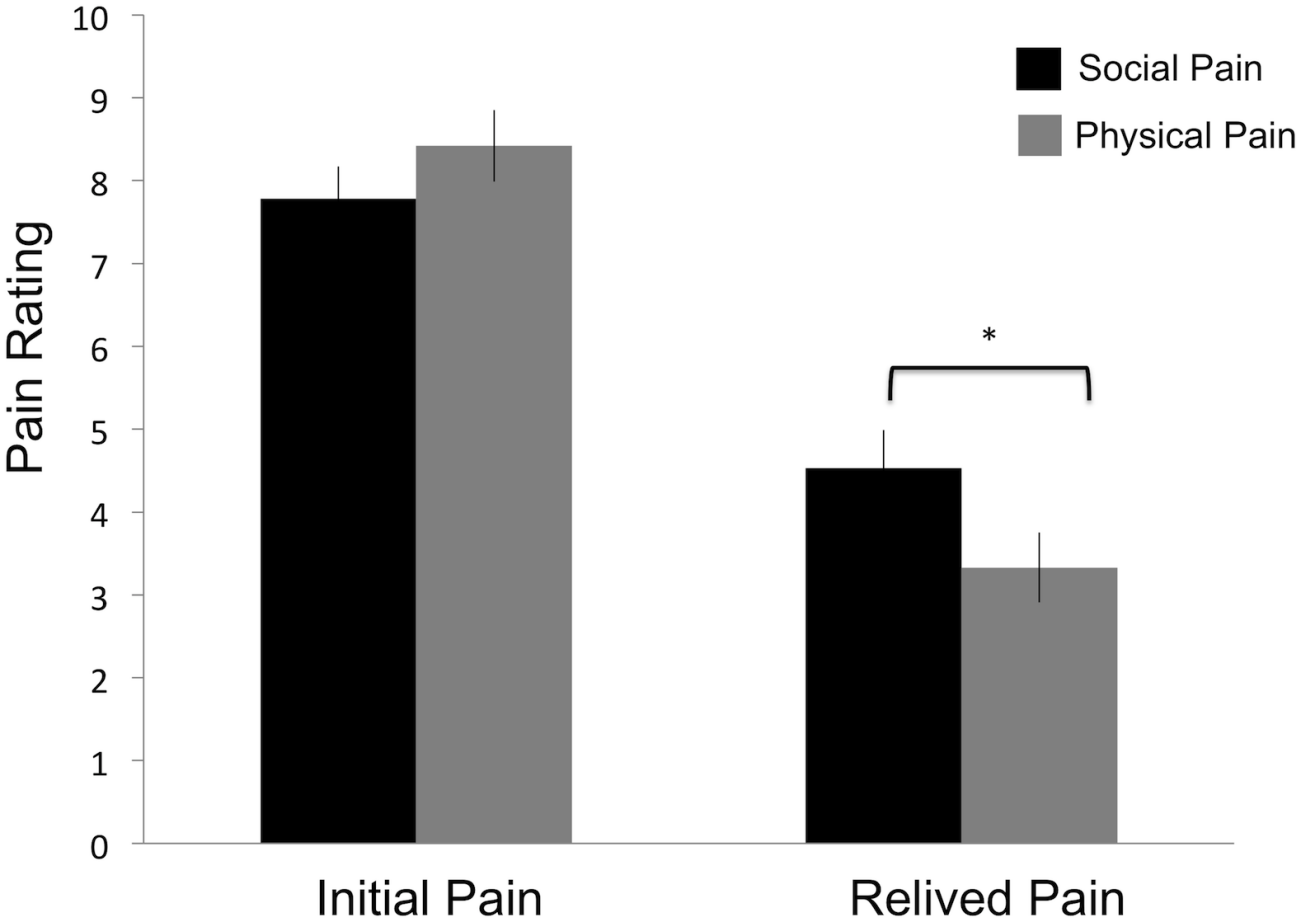
Self-reported pain ratings for the initial and relived pain.

Replicating past work [3], we found a significant interaction between pain type (social versus physical) and time of pain (initial pain versus relived pain) *F*(1,17) = 4.72, *p*<.05, Fig 2. Using a 0–10 scale, participants reported no significant differences in their ratings of initial pain to the social pain event (mean = 7.78, SD = 1.66) vs. physical pain event (mean = 7.94, SD = 2.65; *t*(17) = -.29, *p* = .77), suggesting that there were no differences in how much pain they felt at the time the original physical or social pain event occurred. However, participants rated experiencing significantly more pain when reliving social pain memories (mean = 4.53, SD = 1.96) compared to when reliving physical pain memories (mean = 3.33, SD = 1.79; *t*(17) = 2.9, *p* = .01). Importantly, the observed difference in relived social vs. physical pain cannot be explained by differences in the amount of time that had passed since the social and physical pain events. A paired sample t-test showed that the mean temporal distances, in months, between the experiment and the initial pain experiences (mean social pain temporal distance = 25 months; mean physical pain temporal distance = 20 months) were not significantly different from each other (*p* = .37).

### Reliving social pain preferentially engages affective pain regions

**Fig 3:**
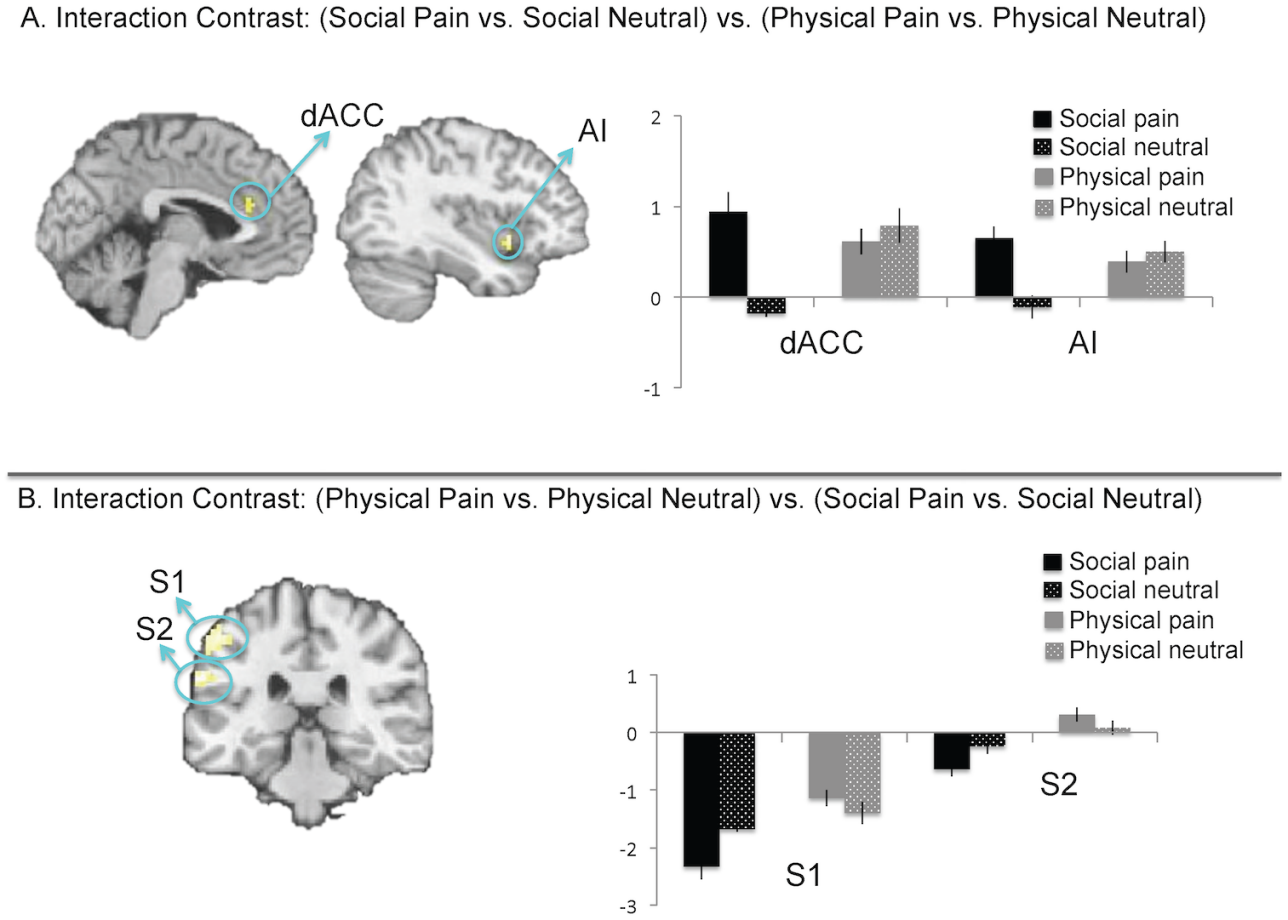
Interaction of reliving social pain vs. social neutral, relative to reliving physical pain vs. physical neutral.

We hypothesized that this greater capacity to re-experience social pain may occur, in part, because individuals recruit greater affective pain system activity while reliving social pain than while reliving physical pain. Consistent with this prediction, the interaction contrast comparing neural activation during social pain reliving vs. social neutral reliving, relative to physical pain reliving vs. physical neutral reliving (i.e., (social pain reliving>social neutral reliving)>(physical pain reliving>physical neutral reliving)), revealed neural activation in affective pain regions (dACC, AI, Fig 3, Table 1) but no sensory pain region activity. This interaction contrast is a highly specific contrast, as it partials out any activity during social and physical pain reliving that might be tied to the content of the pain type (that is, cognition related to social versus physical processing). Post-hoc analyses of the interaction revealed greater activity in the dACC and AI clusters during social pain reliving relative to social neutral reliving (*p*s <.001), but no differences in activity during physical pain reliving relative to physical neutral reliving (*p*s >.14). Importantly, there was also greater activity in the dACC and AI during social pain reliving relative to physical pain reliving (*p*s<.05).

**Table 1:** Brain regions showing increased activation from the factorial design contrasts: (social pain reliving>social neutral reliving) vs. (physical pain reliving>physical neutral reliving); (physical pain reliving>physical neutral reliving) vs. (social pain reliving>social neutral reliving) vs. (physical pain reliving>physical neutral reliving); social pain reliving versus social neutral reliving; social pain reliving versus physical pain reliving; physical pain reliving versus physical neutral reliving; and physical pain reliving versus social pain reliving.

**(Social Pain Reliving > Social Neutral Reliving) vs. (Physical Pain Reliving > Physical Neutral Reliving)**						
Region	Laterality	x	y	z	*t*	K
dACC	L	-6	27	18	2.97	12
Anterior Insula	L	-33	12	-9	3.62	11
**(Physical Pain Reliving > Physical Neutral Reliving) vs. (Social Pain Reliving > Social Neutral Reliving)**						
Region	Laterality	x	y	z	*t*	K
Primary Somatosensory Cortex	L	-48	-33	57	3.2	49
		-45	-33	45	3.11	
		-57	-27	45	3.1	
Secondary Somatosensory Cortex	L	-63	-33	27	3.18	24
**Social Pain Reliving > Social Neutral Reliving**						
Region	Laterality	x	y	z	*t*	K
dACC	L	-3	33	21	2.86	12
AI	L	-33	12	-9	3.92	23
	L	-27	15	-15	2.86	-
**Social Pain Reliving > Physical Pain Reliving**						
Region	Laterality	x	y	z	*t*	k
DMPFC	L	-6	54	21	3.02	12
	R	3	33	15	3.1	10
dACC	L	-3	36	9	2.89	-
**Physical Pain Reliving > Social Pain Reliving**						
Region	Laterality	x	y	z	*T*	k
Inferior Frontal Gyrus	L	-45	39	12	4.91	72
	L	-39	45	9	4.55	-
Somatosensory Cortex	L	-57	-30	33	6.24	612
	L	-57	-30	42	6.22	-
	L	-63	-21	18	4.98	-
	R	33	-42	69	4.16	392
	R	57	-33	54	3.98	-
	R	66	-27	39	3.94	-

**Fig 4:**
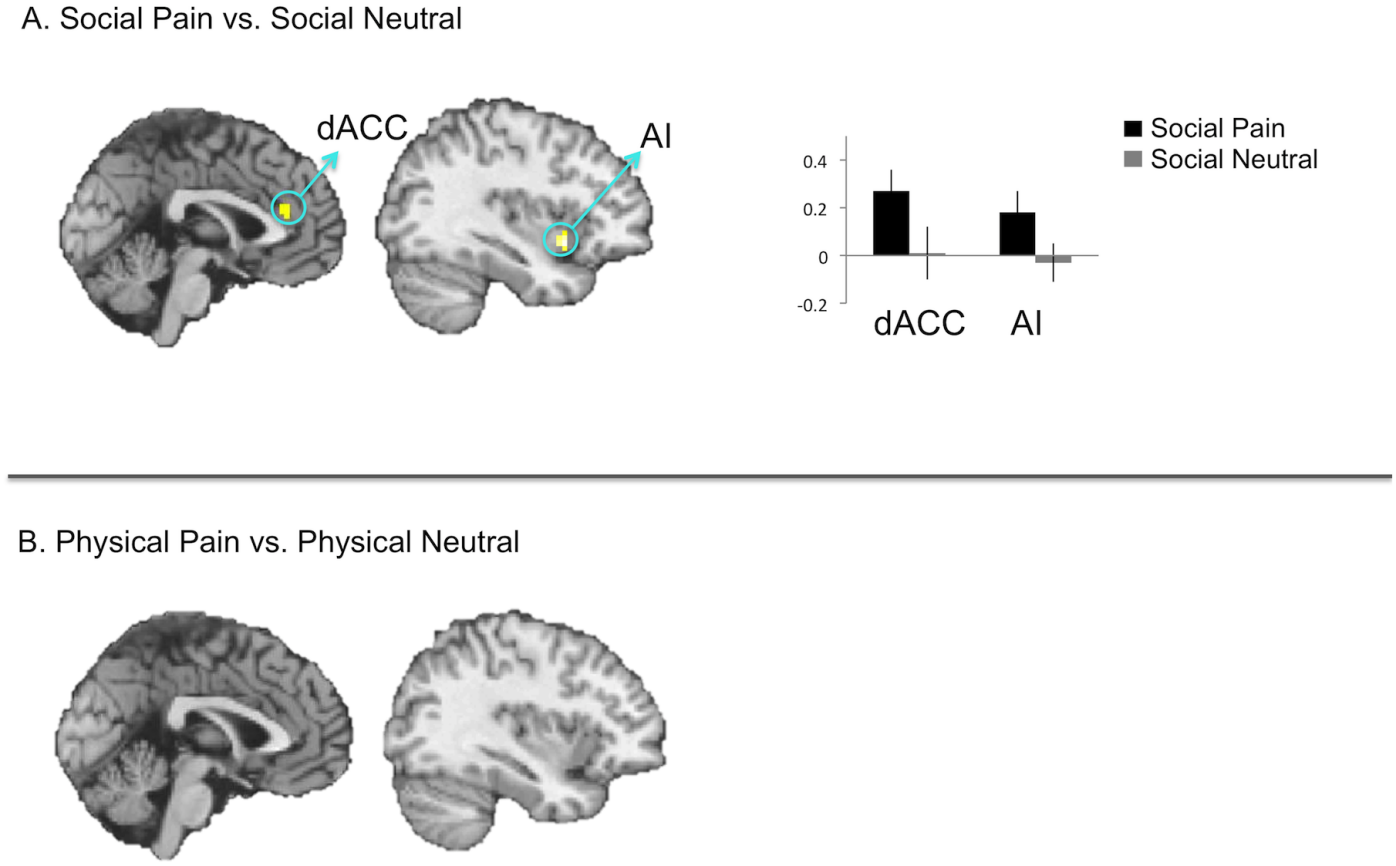
A. Direct comparisons of reliving social pain (vs. social neutral). B. Direct comparisons of reliving physical pain (vs. physical neutral). The blank brain indicates that no significant differences were observed in the reliving physical pain (vs. physical neutral) contrast.

Moreover, direct comparisons of each reliving pain condition to its tailored control condition (social pain vs. social neutral reliving; physical pain vs. physical neutral reliving) showed similar results (Fig 4A and 4B, Table 1). Affective pain regions were significantly more active when reliving social pain (vs. social neutral) memories, but not significantly more active when reliving physical pain (vs. physical neutral) memories.

The reverse interaction contrast comparing neural activation during physical pain reliving vs. physical neutral reliving, relative to social pain reliving vs. social neutral reliving (i.e., (physical pain reliving>physical neutral reliving)>(social pain reliving>social neutral reliving)), revealed activation in primary and secondary somatosensory cortex (S1/S2)) (Fig 3, Table 1), but no activity in affective pain regions. Post-hoc analyses for the S1 and S2 clusters revealed greater S1 and S2 activity during physical pain vs. social pain reliving (*p*s<.001) and less activity in S1 and S2 for social pain vs. social neutral reliving (*p*s<.001). The S1 and S2 clusters were not significantly more active for physical pain reliving vs. physical neutral reliving (S1 p = .18; S2 *p* = .10).

Similarly, direct comparisons of each reliving pain condition to its tailored control condition (physical pain vs. physical neutral reliving; social pain vs. social neutral reliving) revealed no significant activation in the direct comparison of reliving physical pain vs. physical neutral memories within the masked search space (although see below for a portion of IFG observed in this contrast outside of the masked search space). However, this may be due to the fact that both the physical pain and physical neutral conditions led to similar patterns of neural activity. Indeed, comparing each physical reliving condition to implicit baseline confirmed that both conditions significantly engaged the IFG and somatosensory regions within the masked search space. Reliving physical pain memories (vs. implicit baseline) significantly activated IFG [x = -39 y = 45 z = 3] and S1/S2 [x = -63 y = -36 z = 30; x = 66 y = -36 z = 33]. Reliving physical neutral memories (vs. implicit baseline) also activated IFG [x = -45 y = 36 z = 0] and S1/S2 [x = 48 y = -18 z = 60; x = 66 y = -36 z = 36] as well as posterior insula [x = 42 y = -18 z = 3]. Thus, reliving physical pain and physical neutral memories may both engage the somatosensory system. In contrast, the direct comparison of social pain reliving (vs. social neutral reliving) showed significant reductions in somatosensory cortex [x = -60 y = -27 z = 45; x = 51 y = -30 z = 48]).

**Fig 5:**
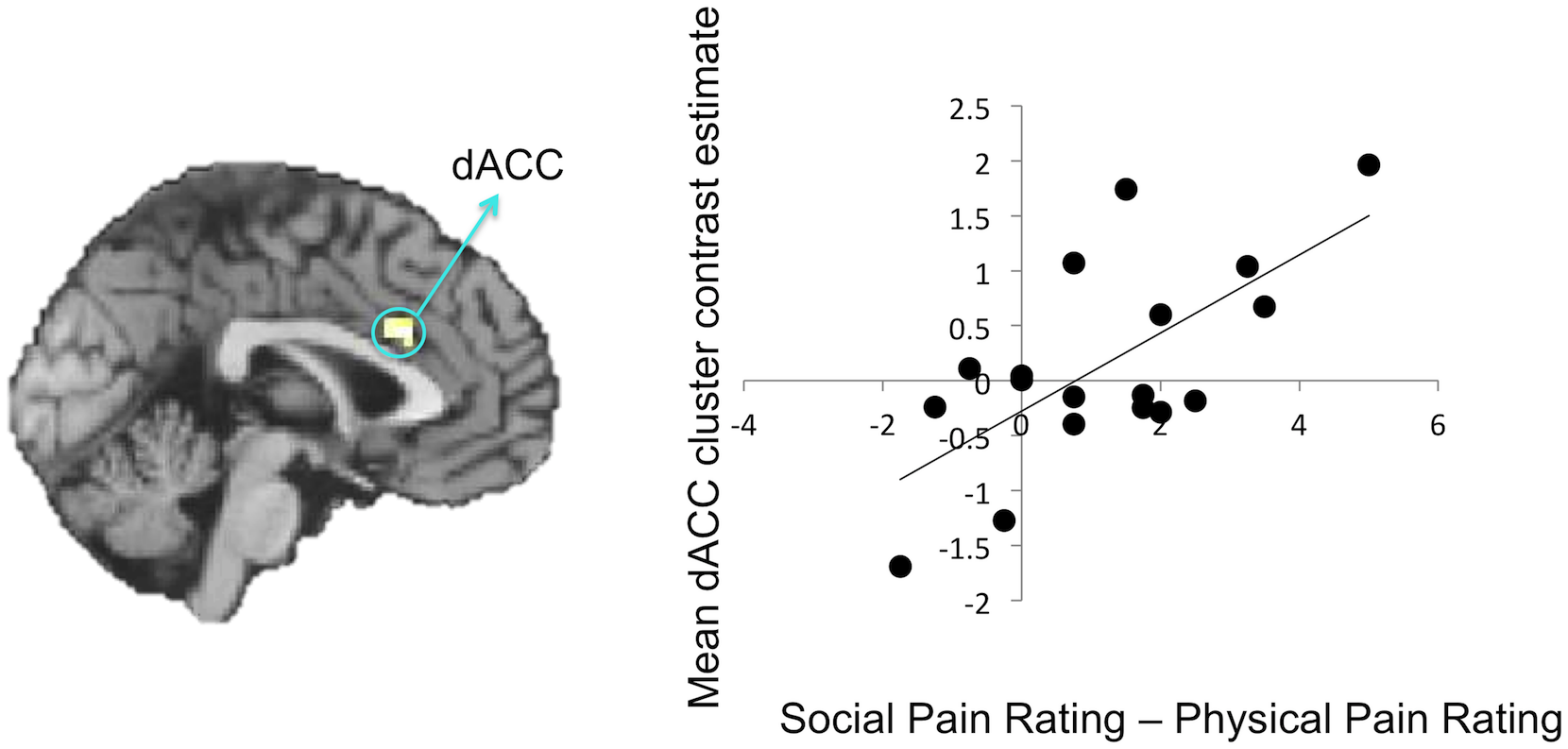
Results from the regression analysis with the difference score in social versus physical pain ratings regressed on the contrast comparing social pain reliving to physical pain reliving. Greater self-reported pain when reliving social vs. physical pain memories correlated with greater activity in the dACC.

Finally, consistent with the findings that relived social pain led to greater self-reported pain and greater affective pain-related activity, a regression analysis revealed that greater pain ratings during social pain vs. physical pain correlated only with greater activation in the dACC (Fig 5, Table 2), suggesting that the bias to feel more relived social pain is reflected in greater activity in this region associated with affective pain system responding.

**Table 2:** Regression results. Regions that correlate positively with self-reported pain during social vs. physical pain reliving.

Regions that correlate positively with self-reported pain during social vs. physical pain reliving.							
Region	Laterality	x	y	z	*t*	k
dACC	L	-3	21	27	3.54	23
	L	-6	27	27	3.47	-

### Different neurocognitive pathways for reliving social and physical pain

Although we observed preferential engagement of the affective pain system during social versus physical pain reliving, this result still begs the question as to why social pain reliving would correspond with greater affective pain system responding, particularly since participants reported experiencing equivalent amounts of pain at the time of the events. To test the possibility that preferential affective pain system responding during social vs. physical pain reliving may result from recruiting different top-down cognitive processes (mental state versus body state processing) during the forms of reliving, we first examined the extent to which participants’ pain memories relied on mental state and bodily state processing.

Consistent with predictions, we observed a two-way interaction between the factors mental state processing vs. body state processing and social pain memory vs. physical pain memory, *F*(1,17) = 203.56, *p*<.0001, such that participants’ social pain memories, compared to physical pain memories, were rated as involving more mental state processing (mean social = 5.75, SD = .91; mean physical = 3.57, SD = .92; *t*(17) = 12.11, *p*<.0001), whereas participants’ physical pain memories, compared to social pain memories, were rated as involving more bodily state processing (mean physical = 6.32, SD = .52, mean social = 1.77, SD = .86; *t*(17) = 18.86, *p*<.0001). In line with the suggestion that the mental state processing of social pain memories may induce relived social pain, degree of mental state processing in social pain memories correlated with the degree of relived social pain (r = .54, *p*<.05). In contrast, but consistent with the prediction that body state processing may not strongly induce relived physical pain, degree of physical state processing during physical pain memories was not significantly correlated with degree of relived physical pain (r = .28, *p* = .26).

**Fig 6:**
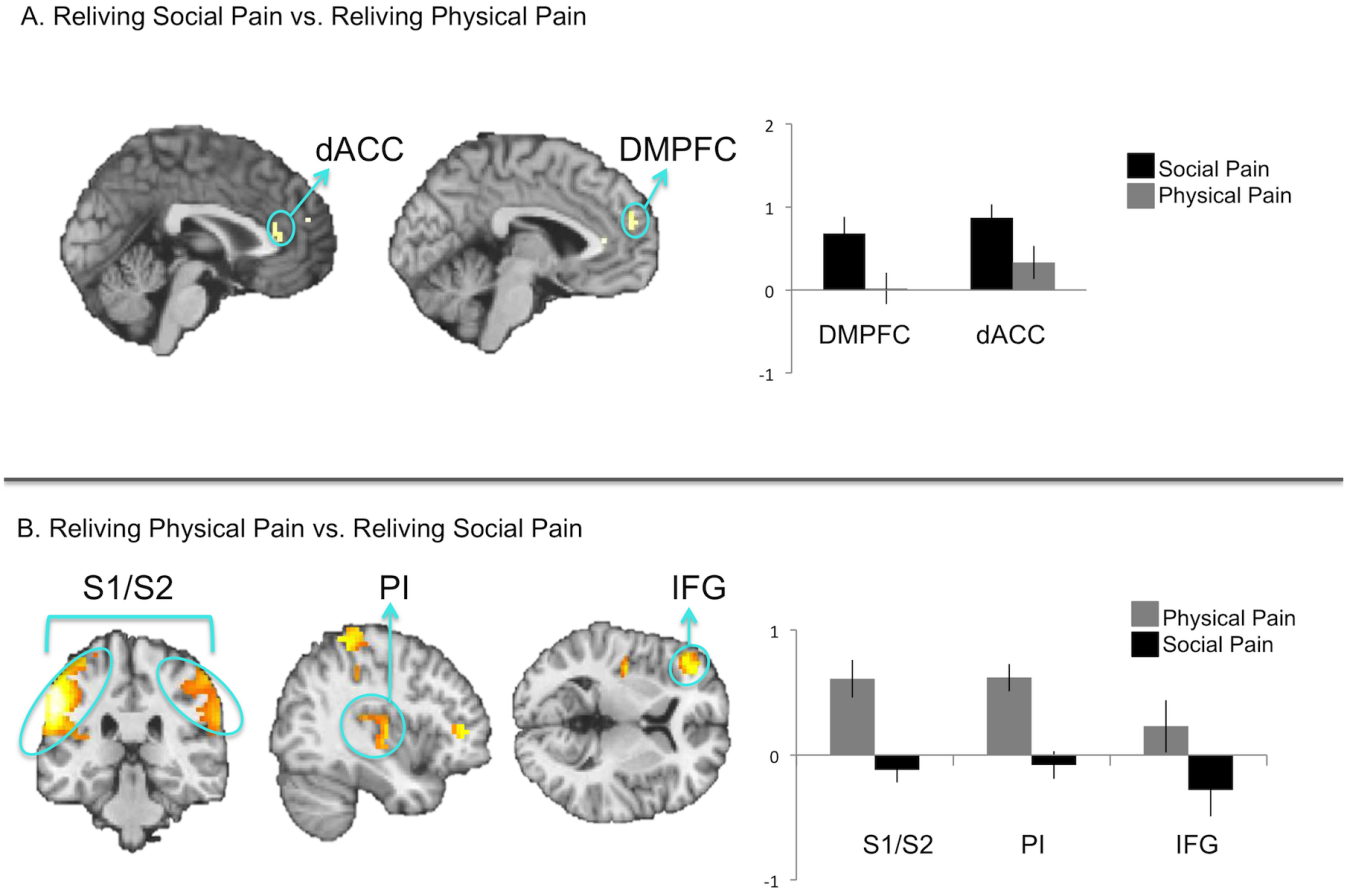
A. Direct comparison of social pain reliving versus physical pain reliving. B. Direct comparison of physical pain reliving versus social pain reliving.

We next performed analyses to further test the idea that, given the different content of the social and physical pain memories, social pain and physical pain reliving may recruit different top-down cognitive processes. First, we directly compared neural activity during social pain reliving with physical pain reliving (Fig 6A, Table 1). These contrasts allowed us to identify activation that is related to the social versus physical aspects of the painful memories (these results could have been masked by the interaction contrasts because these interactions control for the social and physical dimensions by comparing each pain condition to its tailored control condition). Consistent with our predictions, social pain reliving versus physical pain reliving engaged DMPFC in addition to regions associated with the affective component of pain (dACC). By comparison, physical pain reliving versus social pain reliving engaged activation in IFG, as well as regions associated with the sensory-discriminative component of pain (S1, S2, posterior insula; Fig 6B, Table 1). Thus, in addition to differences in affective pain system activation, reliving social and physical pains also differ in the prefrontal mechanisms engaged during the two forms of reliving, with social pain reliving engaging more DMPFC activity and physical pain reliving engaging more IFG activity. Because these contrasts do not account for the tailored baseline conditions, these results simply highlight that reliving each form of pain engages prefrontal mechanisms associated with the social vs. physical content of the memory.

**Fig 7:**
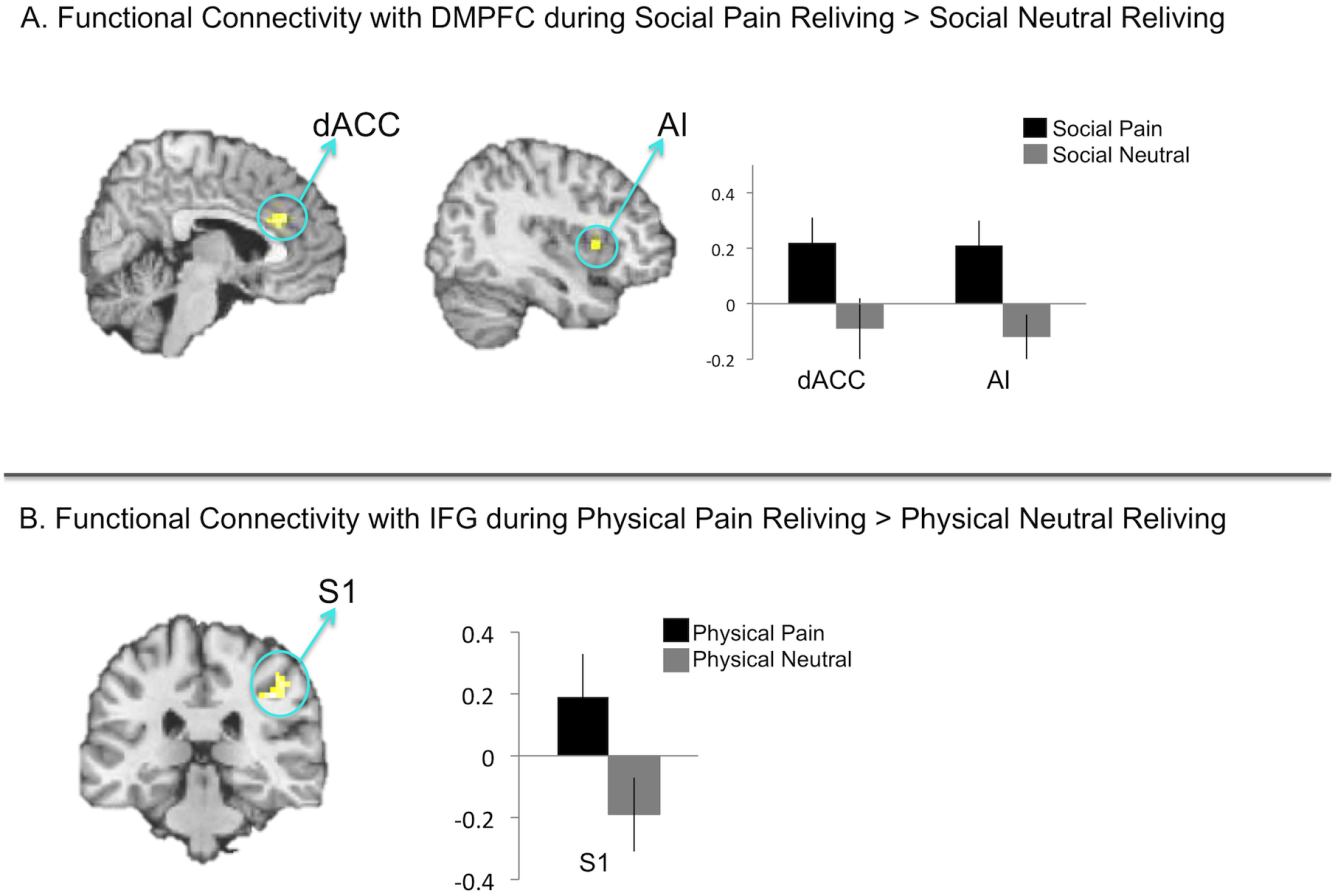
A. Brain regions whose activation was found to be functionally coupled with DMPFC during social pain reliving (relative to reliving neutral social memories). B. Brain regions whose activation was found to be functionally coupled with IFG during physical pain reliving (relative to reliving neutral physical memories).

Second, we performed psychophysiological interaction (PPI) analyses with DMPFC and IFG seed regions to test the possibility that DMPFC functionally correlates with the affective pain system during social pain reliving, whereas IFG does not during physical pain reliving. Activation in the DMPFC during social pain reliving (relative to social neutral reliving) functionally correlated with activity in the dACC and AI (Fig 7A, Table 3) but not with activity in sensory pain regions. Importantly, and consistent with the idea that activity in affective pain regions track self-reported pain distress, activation during social pain reliving in the dACC and AI clusters correlated with participants’ social pain reliving ratings (dACC r = .43, *p*<.05; AI r = .54, *p*<.01). Thus, during social pain reliving, DMPFC appears to functionally relate to affective pain region activation, and these latter regions contribute to the phenomenology of enhanced relived pain.

**Table 3:** Brain regions showing increased functional connectivity with DMPFC during social pain reliving versus social neutral reliving and IFG during physical pain reliving versus physical neutral reliving.

**Social Pain Connectivity**							
	Region	Laterality	x	y	z	*t*	k
	dACC	R	9	30	24	3.74	30
		R	0	36	24	3.6	-
	Anterior Insula	R	33	15	6	3.95	15
		R	42	15	3	3.2	-
**Physical Pain Connectivity**							
	Region	Laterality	x	y	z	*t*	k
	Primary Somatosensory Cortex	R	42	-30	39	3.14	20
			48	-30	45	3.44	-

In contrast, a PPI analysis examining which regions’ activation correlated with IFG during physical pain reliving (relative to physical neutral reliving) revealed one cluster in S1 (Fig 7B, Table 3) but no affective pain regions. Consistent with the idea that sensory-related neural regions are not as strongly correlated with self-reported pain distress, the correlation between activation in this S1 cluster from the functional connectivity analysis was not significantly correlated with relived physical pain ratings (r = .26, *p* = .29).

### Whole-brain analyses

Finally, we followed up our masked results by searching across the whole brain. The following clusters appeared in addition to the ones that were observed in the masked analyses (See S1 and S2 Tables). In the interaction contrast comparing: (social pain reliving vs. social neutral reliving) vs. (physical pain reliving vs. physical neutral reliving), we observed additional clusters in regions associated with mental state processing (tempoparietal junction (TPJ), middle temporal gyrus, and temporal pole). In the social pain versus social neutral contrast, a cluster in the thalamus was also found. The physical pain vs. physical neutral comparison revealed a posterior portion of IFG that was outside of the masked region. In the contrast directly comparing social pain vs. physical pain, we observed activity in ventromedial prefrontal cortex (VMPFC) and precuneus (PCC). Additional clusters also appeared in the social pain versus social neutral PPI analysis in hypothalamus, pons, and parahippocampal gyrus. These whole-brain results should be interpreted with caution, since the whole-brain search threshold was relatively liberal.

## Discussion

Research showing that negative social experiences recruit the affective pain system and the corresponding surprising consequences (e.g., Tylenol has been shown to reduce feelings of social pain [41]) has garnered great interest in the past decade of social neuroscience research. Yet, these forms of pain differ in important facets of phenomenological experience [3, 42] and in specific types of computational subprocesses [12], and addressing these distinctions may prove equally informative. Specifically, the goal of the present study was to better understand the well-documented, but poorly understood, phenomenon that humans are more easily able to relive past social pains than past physical pains [3].

Replicating past behavioral findings [3], in an fMRI scanning environment we showed that participants reported more pain in response to reliving social (vs. physical) pain memories. Reliving social (vs. physical) pain also more strongly activated brain regions associated with the affective component of pain (dACC, AI), and activation in the affective pain regions during reliving correlated with self-reported relived pain. Moreover, greater affective pain system responding when reliving social (vs. physical) pain may be due, in part, to the recruitment of different top-down neurocognitive mechanisms to generate the two forms of pain. Social pain reliving recruited DMPFC, a region commonly associated with mental state processing [11–13], and this region functionally communicated with affective pain regions (dACC and AI) during social pain reliving. In contrast, reliving physical pain showed functional communication between IFG, a region commonly associated with retrieving information about the body [19–25], and the somatosensory cortex, associated with the sensory-discriminative component of pain.

These results speak to the interesting phenomenological differences between social and physical pain memories. For example, in one study, participants recalled a past physical pain event and were asked to rate their memories along several dimensions [5]. Interestingly, none of the participants endorsed the question: “when you thought about the pain did you re-experience it (have the experience of being in pain again)?” and 41% of subjects were unable to even recall the sensory quality of the pain. In contrast, Chen et al. [3] found that people easily re-experience a social pain that occurred up to five years prior, and re-experience this social pain significantly more than a physical pain matched on intensity at the time of the event [3].

One possibility for this difference, suggested by Morley [5], is that the intensity vs. distress of a painful experience is retrieved from memory via different mechanisms. Consistent with this suggestion, we observed that reliving social pain engaged a DMPFC-affective pain system pathway, whereas reliving physical pain engaged an IFG-sensory pain system pathway. Interestingly, it was recently found that not only does thinking about *past* social pains (vs. *past* physical pains) generate more pain in the present, but also imagining *future* social pain (vs. *future* physical pain) leads to more pain in the present [42]. The region of DMPFC observed in our study is also associated with mental simulation of future events and prospective memory [43]. Thus, it is possible that both reliving past social pain and imagining future social pain commonly engage DMPFC-affective pain system connectivity to magnify internally induced social pain.

Consistent with the idea that different pain system activity during social and physical pain reliving may reflect different neurocognitive pathways to induce reliving, we observed that social and physical pain memories emphasized different types of information-processing: social pain memories emphasized the mental states associated with the pain whereas physical pain memories emphasized the physical bodily states associated with the pain. Accordingly, social pain reliving recruited DMPFC, a region reliably associated with mental state processing [13, 14, 44], and this DMPFC activation functionally correlated with enhanced affective pain system responding during social pain reliving. In contrast, reliving physical pain recruited IFG, a region reliably associated with thinking about bodily states [22, 23], and this IFG activation functionally correlated with enhanced somatosensory activity. Thus, DMPFC may contribute to relived social pain via its communication with affective pain regions. IFG, on the other hand, may be involved in increasing the sensory-discriminative component of pain, but without coordinating activation in affective pain regions and hence not easily increasing feelings of pain.

Indeed, the DMPFC-affective pain system pathway observed here during social pain reliving may also help explain other affective phenomena in which more or less mental state processing corresponds with more or less painful feelings. For example, past work has shown that physical and emotional pain caused by understanding another person’s intention to harm hurts more than the same pain resulting from non-intentional causes [45, 46]. In fact, while over time participants habituate to randomly delivered painful shocks, they do not habituate to the pain caused by another person’s intention to hurt them [45]. Understanding that your pain was caused by another person’s intentions requires mental state processing, and thus may elicit affective pain system responding via DMPFC, and connectivity between these regions may contribute to the sustained pain over time.

In addition to providing a potential neural mechanism guiding why mental state processing corresponds with enhanced affective pain, our results contribute to a growing literature implicating DMPFC in social cognitive memory. DMPFC has recently been shown to sustain social cognitive information in working memory [47], retrieve social cognitive facts from semantic memory [48], and even support the memory benefit for socially encoded information [49]. Our results add to this literature by showing that DMPFC may also specifically contribute to social-emotional autobiographical memory.

While the finding that relived social pain can activate affective pain regions is consistent with prior research [7], it is noteworthy that the comparison of physical pain reliving vs. physical neutral reliving showed no significant differences in pain-related neural activation within our anatomical masks (although we did observe clusters outside of our masked search space at more liberal statistical thresholds). With regard to sensory-related activation, this lack of significant differences may reflect the possibility that the IFG and somatosensory cortex are equally engaged when reliving past painful and neutral physical memories. Indeed, IFG has been shown to activate during painful and neutral physical memory processes [11, 50] [10, 27] and to the extent that people can relive sensory experiences, the somatosensory system may also equally engage during these two forms of reliving. Alternatively, it is possible that the experimental design and sample size used in this study were not ideal for detecting real differences in neural activation between reliving physically painful and neutral memories. Future research with larger datasets may help determine if these two forms of reliving can be distinguished at the neural level of analysis.

With regard to affective-related activation, our lack of significant dACC and AI activity during physical pain memory reliving is consistent with a past study examining retrieval of physically painful vs. physically neutral memories [11]. Nonetheless, the results are seemingly in contrast with two different pain memory fMRI studies, one of which observed AI [10] and the other dACC [27] in physical pain memory paradigms. Albanese et al. [10] found AI, but not dACC, activated when participants discriminated, after a short delay period (6 seconds), whether a second pain stimulus was stronger or weaker than an initial pain stimulus. However, we suspect this AI result speaks to what participants *can do* during a pain memory paradigm, rather than speaking to the phenomenology of reduced physical pain re-experiencing in the real world. That is, the result may reflect the fact that participants were explicitly instructed to make physical pain discriminations. Thus, people may be able to activate AI when instructed to perform a task for which performance improves by maintaining affective pain representations in working memory. However, this does not speak to whether this AI activation is related to ‘re-experiencing’ or ‘reliving’ the previous pain, nor whether participants spontaneously engage AI when they consider their autobiographical painful memories. In fact, in the Kelly et al. [27] study, when participants considered their autobiographical pain memories, no AI activity was observed. Instead, dACC activated when participants retrieved autobiographical memories in response to pain words, such as ‘hurt’ [27]. However, in the Kelley et al. study [25], social and physical pain memories were not distinguished and participants reported retrieving memories with both ‘physical’ and ‘affective’ associations. Thus, it is not clear that only physical pain memories were retrieved, particularly since past work shows people often associate pain-related words with negative social experiences [51]. Taken together, paradigm differences between our own study and those of Albanese et al. [10] and Kelley et al. [27] may explain differences in the physical pain memory findings.

### Limitations

It is noteworthy that social and physical pain may differ on several dimensions in addition to mental state vs. bodily state processing [12]. For example, social and physical pains may tend to differ in the discrete versus ongoing nature of the initial pain duration (e.g., the difference between a brief but painful needle injection vs. a slow but painful break-up), and/or, potentially, the mechanisms engaged during the encoding of the event (e.g., presence or absence of noxious input). It is also possible that social pains remain ‘open wounds’ for longer durations than certain physical pains. Given that participants’ social pains in this study occurred on average 25 months prior to their scan, it is unlikely that participants continued to experience their former social pains as though they were occurring in the present. Nonetheless, future research is needed to examine the extent to which subjects continue to experience past social pains as present pains and whether this affects participants’ ability to relive these experiences.

Similarly, it is possible that the painful feelings accompanying physical pain cannot occur without the presence of concurrent externally generated sensory stimulation, whereas social pain can. Though not directly tested in the present study (e.g., we did not compare live social vs. physical pain with relived social vs. physical pain), our results are consistent with this interpretation. Moreover, reliving a socially painful event could lead to other affective experiences besides pain, such as feelings of sadness, loss, or even anger. Hence, the neural activity observed during social pain reliving may not be specific to painful feelings, but may also include these other emotional components as well. However, given that people use similar types of pain words to describe both experiences of social and physical pain [46] and given that similar neural regions are activated in response to the experience of both types of events (though they may rely on different neural computations; [10]), it is still noteworthy that these affective experiences do not seem to be induced to the same extent when reliving a physically painful event relative to a socially painful event.

## Conclusion

Our results elucidate possible neural mechanisms that may explain why social pain is more easily relived than physical pain. Social pain reliving (vs. physical pain reliving) more strongly engaged the affective pain system, which correlated with self-reported pain. In contrast, physical pain reliving (vs. social pain reliving) more strongly engaged the sensory-discriminative pain system, and activation in this system did not track with self-reported pain. Different patterns of pain system responding between the two forms of reliving may be due, in part, to the recruitment of different top-down neurocognitive pathways to internally generate the relived pain: a medial frontoparietal-affective pain system pathway may support enhanced relived social pain, whereas a lateral frontoparietal-sensory pain system pathway may support the relatively reduced relived physical pain. These pathways help explain otherwise perplexing observations of enhanced social pain and reduced physical pain during reliving. And perhaps most importantly, the pathways underscore the value of a broader theoretical framework of social and physical pain, that accounts for not only the similarities, but also differences, guiding these two forms of suffering.

## Supporting Information

S1 Fig (TIFF)

S1 Table (PDF)

S2 Table (PDF)
