# Supplementary figures and images for: Why Social Pain Can Live on: Different Neural Mechanisms Are Associated with Reliving Social and Physical Pain
Source: PLoS One. 2015 Jun 10;10(6):e0128294. doi: 10.1371/journal.pone.0128294 (PMC4465485; doi:10.1371/journal.pone.0128294)

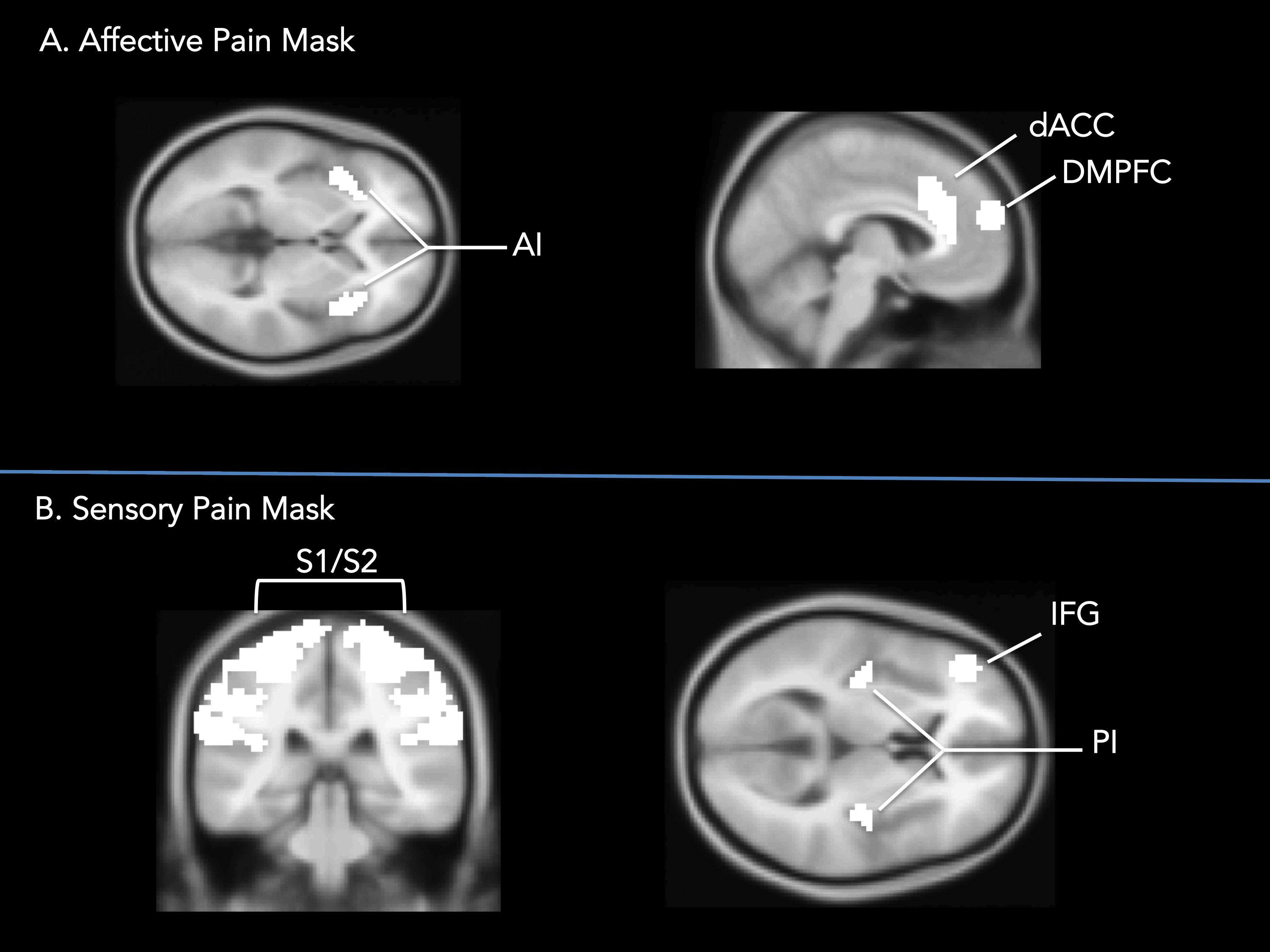

Supplement: S1 Fig — (TIFF) [file pone.0128294.s001.tiff]
