# Supplementary material for: Why Social Pain Can Live on: Different Neural Mechanisms Are Associated with Reliving Social and Physical Pain
Source: PLoS One. 2015 Jun 10;10(6):e0128294. doi: 10.1371/journal.pone.0128294 (PMC4465485; doi:10.1371/journal.pone.0128294)
Supplement: S1 Table — (PDF) [file pone.0128294.s002.pdf]

S1 Table. Whole-brain results. Brain regions showing increased activation from the factorial design contrasts: (social pain reliving>social neutral reliving) vs. (physical pain reliving>physical neutral reliving); (physical pain reliving>physical neutral reliving) vs. (social pain reliving>social neutral reliving) vs. (physical pain reliving>physical neutral reliving); social pain reliving versus social neutral reliving; social pain reliving versus physical pain reliving; physical pain reliving versus physical neutral reliving; and physical pain reliving versus social pain reliving.

**(Social Pain Reliving > Social Neutral Reliving) vs. (Physical Pain Reliving > Physical Neutral Reliving)**						
Region	Laterality	x	y	z	*t*	k
dACC	L	-6	27	18	2.97	12
Anterior Insula	L	-33	12	-9	3.62	15
Middle Temporal Gyrus	L	-57	-18	-18	3.26	18
Temporal Pole	L	-48	9	-24	3.61	13
Tempoparietal Junction (TPJ)	L	-48	-60	33	3.12	22
**(Physical Pain Reliving > Physical Neutral Reliving) vs. (Social Pain Reliving > Social Neutral Reliving)**						
Region	Laterality	x	y	z	*t*	k
Somatosensory Cortex	L	-57	-27	45	3.1	149
Inferior Frontal Gyrus (IFG)	R	39	51	-6	3.41	60
Inferior Frontal Gyrus (IFG)	R	48	45	6	3.18	-
Fusiform Gyrus	L	-33	-54	-15	2.97	15
Precentral Gyrus	L	-60	6	21	3.29	29
Precentral Gyrus	L	-54	6	39	2.99	-
Supramarginal Gyrus	L	-63	-33	27	3.18	25
**Social Pain Reliving > Social Neutral Reliving**						
Region	Laterality	x	y	z	*t*	k
DMPFC	L	-3	51	27	3.48	36
dACC	L	-3	33	21	2.86	14
Anterior Insula	L	-33	12	9	3.92	28
	L	-27	15	-15	2.86	-

Thalamus	L	-3	-6	3	3.17	18
Cerebellum	R	24	-81	-30	3.28	32

Social Pain Reliving > Physical Pain Reliving

Region	Laterality	x	y	z	*t*	k
DMPFC	R	6	51	21	3.59	34
	L	-6	54	21	3.02	-
dACC	R	3	33	15	3.1	11
	L	-3	36	9	2.89	-
MPFC	R	12	39	0	3.38	26
VMPFC	R	3	51	-21	3.71	19
subACC	R	12	39	0	3.38	26
	R	9	48	3	3.06	-
Thalamus	R	9	-6	-12	3.72	45
	R	0	-6	0	3.04	-
Brainstem	R	15	-24	-21	3.35	20
Precuneus	R	9	-51	30	4.31	164
	L	-6	-57	36	4.19	-
	L	-12	-51	15	3.19	-
Cerebellum	R	27	-81	-30	3.5	31

Physical Pain Reliving > Physical Neutral Reliving

Region	Laterality	x	y	z	*t*	k
posterior IFG	L	-30	33	3	3.65	41
	L	-36	27	6	3.48	-
Occipital Lobe	R	39	-81	-15	3.13	10

Physical Pain Reliving > Social Pain Reliving

Region	Laterality	x	y	z	*t*	k
Inferior Frontal Gyrus	R	48	48	3	5	310
	L	-48	39	15	5.01	468
	R	54	9	15	3.3	115
Primary Somatosensory Cortex	R	36	-42	69	4.2	1193
	L	-57	-30	42	6.22	2632
Superior Frontal Gyrus	L	-15	-3	-69	3.71	244
Precentral Gyrus	R	60	12	33	4.34	115
Inferior Temporal Cortex	L	-42	-54	-9	4.46	477
	R	60	-48	-18	4.15	98
