# Supplementary material for: Why Social Pain Can Live on: Different Neural Mechanisms Are Associated with Reliving Social and Physical Pain
Source: PLoS One. 2015 Jun 10;10(6):e0128294. doi: 10.1371/journal.pone.0128294 (PMC4465485; doi:10.1371/journal.pone.0128294)
Supplement: S2 Table — (PDF) [file pone.0128294.s003.pdf]

S2 Table. Whole-brain results. Brain regions showing increased functional connectivity with DMPFC during social pain reliving versus social neutral reliving and IFG during physical pain reliving versus physical neutral reliving

Social Pain Connectivity

Region	Laterality	x	y	z	*t*	k
dACC	R	12	27	21	4.8	40
Anterior Insula	R	33	15	6	3.95	23
ParahippocTa.ampal Gyrus	L	-24	-45	-9	3.5	51
Hypothalamus	L	-3	-12	-6	4.03	41
Pons	L	-6	-24	-24	4.25	41
Caudate	L	-15	15	57	5.73	277
Precuneus	R	15	-54	57	4.5	44
Posterior Cingulate	R	12	-27	39	4.68	37
Tempoparietal Junction (TPJ)	R	42	-69	18	4.66	90
posterior Superior Temporal Gyrus (pSTS)	R	57	-18	-9	4.11	61
Precentral Gyrus	L	-45	-6	6	4.43	135
Occipital Lobe	L	-39	-84	9	4.98	37
Occipital Lobe	R	33	-81	12	4.7	47

Physical Pain Connectivity

Region	Laterality	x	y	z	*t*	k
Primary Somatosensory Cortex	R	42	-30	39	3.72	21
		48	-30	45	3.44	-
